# Aerobic physical exercise versus dual-task cognitive walking in cognitive rehabilitation of people with stroke: a randomized clinical trial

**DOI:** 10.3389/fpsyg.2023.1258262

**Published:** 2023-10-13

**Authors:** Reinaldo Maeneja, Cláudia R. Silva, Inês S. Ferreira, Ana Maria Abreu

**Affiliations:** ^1^Institute of Health Sciences, Universidade Católica Portuguesa, Lisbon, Portugal; ^2^Faculdade de Ciências da Saúde e Desporto, Universidade Save, Maxixe, Mozambique; ^3^Escola Superior de Saúde de Alcoitão, Alcabideche, Portugal; ^4^Faculty of Health Sciences, Universidade Europeia, Lisbon, Portugal; ^5^Center for Research in Neuropsychology and Cognitive and Behavioral Intervention (CINEICC), Faculdade de Psicologia e de Ciências da Educação (FPCE), Universidade de Coimbra, Coimbra, Portugal; ^6^Psychological Assessment and Psychometrics Laboratory (PsyAssessmentLab), Faculdade de Psicologia e de Ciências da Educação (FPCE), Universidade de Coimbra, Coimbra, Portugal; ^7^Center for Interdisciplinary Research in Health, Universidade Católica Portuguesa, Lisbon, Portugal

**Keywords:** stroke, exercise, cognition, cognitive-gait, dual-task

## Abstract

**Introduction:**

Stroke is a neurological deficit caused by an acute focal injury to the central nervous system due to vascular injury that can result in loss of neurological function, lasting brain damage, long-term disability and, in some cases, death. The literature reports that aerobic physical exercise, as well as dual-task cognitive walking, are used for the cognitive recovery of people with stroke. We aimed to assess whether aerobic physical exercise influences post-stroke cognitive recovery, namely performance on selective and sustained attention. We tested the hypothesis that post-stroke aerobic physical exercise leads to more significant gains than post-stroke dual-task cognitive walking.

**Methods:**

We used a Randomized Clinical Trial, single-blind, parallel group, to verify the existence of differences between two groups. A total of 34 patients with subacute to chronic stroke were divided into two groups to train three times a week for 12 weeks: the aerobic physical exercise (PE) group engaged in 20 min on a treadmill, 20 min on a stationary bicycle and 5 min on a desk bike pedal exerciser per session; the dual-task (DT) gait exercise group walked for 45 min while simultaneously performing cognitive tasks per session. All participants were assessed on cognitive functioning with the Mini-Mental State Examination (MMSE) and d2 Test of Attention before acute interventions and post interventions. We have also applied a Visual Analog Scale to monitor the participants’ perceived difficulty, pre-, post-acute, and post-chronic interventions. Participants also responded to a Borg Scale of perceived exertion following the acute and the final session of chronic training.

**Results:**

A mixed model ANOVA revealed a significant interaction effect with a large effect size for most of the cognitive variables under study. The variables associated with the d2 Test of Attention showed significant differences between the groups, mainly from T0 to T2. Also for MMSE, an ANOVA revealed a significant interaction effect with significant improvements from T0 to T2. Our results strongly suggest that aerobic physical exercise is more beneficial than dual-task cognitive-gait exercise since in the PE group, cognitive attention scores increase, and cognitive impairment and perception of exertion decrease, compared to the DT group.

**Conclusion:**

These findings support that PE provides more significant benefits for patients post-stroke when compared to DT.

## 1. Introduction

Nowadays, physical activity is decreasing in tandem with the demands of contemporary life, contributing to a greater sedentary lifestyle with health implications (Sawalla Guseh et al., 2022). Physical activity is a voluntary activity produced by skeletal muscles that results in energy expenditure, and exercise represents its practice in a structured form (Mandolesi et al., 2018). Physical activity is recommended for healthy people to prevent disease, promote well-being, and for people with sicknesses to improve their health status (Kim et al., 2013; Sánchez-González et al., 2021). There is evidence that participation in regular physical activity reduces the incidence of a wide range of chronic diseases and mental disorders (Posadzki et al., 2020; Zeibig et al., 2023), as well as preventing the onset of neurodegenerative processes (Sánchez González et al., 2018). The benefits of physical activity are wide-ranging, including improved muscle function and cardiorespiratory function and metabolic regulation (Pang et al., 2013; Bangsbo et al., 2015; Zheng et al., 2016), decreased anxiety and depression levels, increased self-esteem (Mandolesi et al., 2018) and social integration (Schüller and Demetriou, 2018). Physical activity also contributes to the optimization of global cognitive functioning (Levin et al., 2017; Sánchez González et al., 2018) since during exercise, cerebral blood flow remains relatively constant, which influences cerebral oxygenation (Rooks et al., 2010; Phillips et al., 2014; Moriarty et al., 2019).

Stroke is a neurological deficit resulting from an acute focal lesion of the central nervous system due to vascular causes, including cerebral infarction, intracerebral hemorrhage, and subarachnoid hemorrhage (Sacco et al., 2013; Ojaghihaghighi et al., 2017). It is known that in there are dozens of interconnected neural systems in the brain, each defined as a circuit of neurons that gives rise to a specific behavior (Cramer et al., 2021). Stroke consists of a breakdown of many neural systems that can lead to motor, perceptual, cognitive, and behavioral problems (Gilmore and Spaulding, 2001). Stroke survivors are conditioned by fatigue, with a prevalence between 38 and 77% (Lerdal et al., 2009; Saunders et al., 2014), a fact that predisposes to a sedentary lifestyle and increased risk of recurrent stroke (Billinger et al., 2014; Saunders et al., 2014). Physical activity thus plays a neuroprotective and essential role in improving the health of stroke survivors (Kramer et al., 2019). In fact, aerobic physical exercise plays an important role in improving aerobic fitness, cardiovascular fitness, walking speed, endurance, balance, and cognitive skills, among other post-stroke health outcomes (Han et al., 2017; Unibaso-Markaida et al., 2019).

Research reports that the mechanisms that have been proposed to justify the relationship between physical activity and cognition include structural elements related to angiogenesis, synaptogenesis, and neurogenesis, mainly through the positive regulation of growth factors (Hötting and Röder, 2013; Cheval et al., 2020; Novak and Ellis, 2021), brain metabolism, neurotransmitters, oxygen availability, glucose regulation and oxidative stress (Marmeleira, 2013). Physical activity also leads to cardiovascular fitness, which, in turn, is accompanied by increased blood flow, which maintains oxygen levels in the brain, an essential element for maintaining the brain metabolism (Komiyama et al., 2017). Physical activity increases adrenaline in the blood, and high levels of adrenaline in the blood are associated with changes in the central nervous system that improve cognitive functions (Brisswalter et al., 2002). Regarding brain-derived neurotrophic factor (BDNF), which is a neurotrophin involved in neural tissue growth and repair (Marston et al., 2017; Lan et al., 2018), studies report that BDNF secretion is enhanced with physical activity and this promotes neuronal survival, increases intracellular calcium levels, facilitates transcription factors, and induces mTOR-mediated mRNA translation for memory consolidation (Loprinzi et al., 2018).

On the other hand, dual task exercises have been encouraged as a type of post-stroke intervention to improve balance, mobility, fall risk and activities of daily living (e.g., Mohammed et al., 2019), and cognition (e.g., Park and Lee, 2019).

Dual tasking is defined as the simultaneous performance of two tasks that can be performed independently, measured separately, and have different objectives (Bayot et al., 2018), such as walking while talking on the phone or while talking to a partner (Agmon et al., 2016). Performing two simultaneous tasks is common in everyday life and represents a highly valuable skill for the individual, and can be considered a prerequisite for a normal life (Fatori et al., 2015; Bayot et al., 2018).

The prefrontal cortex, as well as parietal regions were identified as playing an important role in managing the simultaneous execution of two different tasks, areas associated with attention, verbal fluency, executive functions, working memory, visuospatial (Hars et al., 2014; Yokoyama et al., 2015; Leone et al., 2017; Worringer et al., 2019). A recent study points out that Dual Task gait performance is associated with functional inter-network connectivity between motor and divided attention networks, as well as processing speed (Droby et al., 2022). It has been shown that, dual-task cognitive-gait allows direct training of the brain’s parietal–frontal networks to divide attention and coordinate actions more efficiently (Gregory et al., 2017; Baek et al., 2021b; Yang et al., 2022). Dual-task exercise with cognitive tasks has been shown to benefit cognitive and gait ability after stroke (Kim G. Y. et al., 2014). Since dual-tasking is a crucial ability that impacts the quality of life, dual-task training has been suggested to be a clinically viable intervention for stroke survivors. However, most research finds more significant benefits for motor abilities (e.g., balance ability, gait, and upper limb function) compared to cognitive abilities (Zhou et al., 2021). A possible reason for this is suggested in a recent review on dual-task interference during walking after stroke. This review points to the decrease in performance in one or both tasks due to several possible reasons, the most common being the secondary task requiring internally-driven responses, imposing a more significant cognitive load that competes with the walking motor task (Tsang et al., 2022).

Fundamentally, aerobic physical exercise increases aerobic fitness, which in turn improves brain structure and function as it preserves brain plasticity (Hars et al., 2014), while cognitive training greatly improves selective brain function (Yu et al., 2018; Liao et al., 2019) as it improves attention, memory, and executive dysfunctions (Kounti et al., 2011). Both aerobic physical exercise and dual task gait involve brain structures such as the prefrontal cortex and cerebellum that interact and exchange motor and cognitive information, affording mutual support and inhibition (Kounti et al., 2011; Yokoyama et al., 2015). However, despite reports of improvement in motor skills as a result of dual-task training, a systematic review of 13 studies shows that the beneficial effect of dual-task training on cognitive function was provided by only one of the 13 studies, rendering the cognitive benefits of dual-task training, inconclusive (He et al., 2018).Hence, we enquire if aerobic physical exercise, which also impacts motor performance, might reap greater cognitive benefits as it does not entail interference and has been shown to impact cognition via direct and indirect mechanisms. Indeed, aerobic physical exercise promotes various physiological adaptations, such as angiogenesis and neurogenesis, that positively affect cortical function (Chin et al., 2015; Koch et al., 2020). Despite the literature suggesting both aerobic physical exercise and dual-task cognitive-gait as beneficial for stroke survivors, we hypothesize that post-stroke aerobic physical exercise might lead to greater cognitive gains when compared to performing post-stroke dual-task cognitive-gait, as the constant foot drag produced by aerobic physical exercise on the treadmill provides an increase in gait rhythm and automaticity, and favors cognitive improvement as well as improvement in motor function (Penati et al., 2019). Therefore, with this study, we aim to investigate if aerobic physical exercise and dual task cognitive-gait differently influence the cognitive recovery of patients with stroke, namely in attention and concentration performance. Importantly, this is the first study that compares the impact of an aerobic exercise series with a more traditional dual-task cognitive walking task on cognitive recovery of people with stroke. Moreover, to our knowledge, this is the first study to investigate the relationship between effort perception and exercise type. These elements are very important for the elaboration of an optimized rehabilitation protocol involving people with stroke.

## 2. Materials and methods

### 2.1. Trial design

This randomized controlled trial, used a single-blind, parallel group, took place at Inhambane Provincial Hospital and Chicuque Rural Hospital, both in Inhambane Province, Mozambique. Individuals with acute and chronic ischemic stroke were recruited and randomly selected for: (1) Physical Exercise (PE) or (2) Dual Task (DT) groups.

This manuscript has been prepared in accordance with the Standards of CONSORT 2010 statement: Updated guidelines for reporting parallel group randomized trials (Schulz et al., 2011) and Template for Intervention Description and Replication (Hoffmann et al., 2014).

The Ethics Committee for Health of the local university in Portugal and the National Committee on Bioethics for Health in Mozambique approved the study. All participants provided written informed consent, and the study followed the ethical guidelines of the Declaration of Helsinki for research involving human subjects (World Medical Association, 2013).

### 2.2. Participants

Participants were recruited from rehabilitation centers in Inhambane in Mozambique, and through word of mouth between July 2021 and May 2022.

Eligibility criteria included: patients with subacute to chronic ischemic stroke (Guo et al., 2020; Stinear et al., 2020); age equal to or greater than 40 years; comprehension of the Portuguese language; absence of communication and motor deficits that would limit participation in the study protocol; Mini-Mental State Examination (MMSE) cut-off point ≥19, taking into account the low education level of the participants (Bertolucci et al., 1994; Sasaki et al., 2015; Yeh et al., 2019); and residence less than 50 km from the study site, so as not to compromise assiduity. Of the 57 participants recruited, 12 were excluded: two were under 40 years old; two due to incomprehension of Portuguese; three due to aphasia and absence of communication; one due to motor deficits that limited participation in the protocol; one due to a MMSE score less than 19; and three because they lived more than 50 kilometers from the site where the research was carried out. Of the remaining 45 participants who signed informed consent, four did not attend the study and seven dropped out before randomization, so the final sample totaled 34 participants, aged between 40 and 75 years, including 19 men and 15 women.

#### 2.2.1. Randomization and blinding

Using a stratified randomization technique (Ivers et al., 2012; Broglio, 2018), blinded participants were assigned to one of two groups, with similar entry characteristics, the aerobic physical exercise group (*n* = 17) and the Dual Task gait exercise group (*n* = 17). The stratification took into account the stroke stages (acute x chronic) (Acharya et al., 2013; Mayo et al., 2015). Participants’ ongoing enrollment followed a “first-come, first-served” basis (Kim S. H. et al., 2014; Mayo et al., 2015; Ozdogar et al., 2020).

### 2.3. Experimental procedure

The study was conducted by a rehabilitation team who monitored the participants’ health status, including a doctor responsible for medical follow-up, a speech therapist who assisted in the screening process of participants, three physiotherapists and an occupational therapist responsible for exercise sessions and conventional physiotherapy, a psychologist who applied neuropsychological tests, and physical education finalists who helped supervise the training sessions.

#### 2.3.1. Intervention

All participants performed three exercise sessions over 12 weeks, totaling 36 sessions. The aerobic physical exercise consisted of 20 min on a treadmill, 20 min on a stationary bike, and 5 min on a table bike. Intensity on the treadmill and resistance on the stationary bike and table bike were adapted to cardiovascular performance, i.e., there was a gradual increase according to the participant’s respiratory rate (Boss et al., 2017; Kim and Yim, 2017). The intensity of aerobic physical exercise was monitored by the Borg Rating of Perceived Exertion (Liu-Ambrose et al., 2022). All participants were encouraged to meet their goals, which would translate into overcoming weekly goals on the treadmill and on the stationary bike (self-overcoming) to improve cardiovascular provision (Jin et al., 2013; Danks et al., 2014). To ensure the physical safety of the participants, two physical education finalists positioned themselves on each side of the treadmill to supervise the training, increase or decrease the intensity according to physical conditioning, and ensure that the participants did not fall. During training both on the ergometric bicycle and on the table bicycle, to strengthen adherence to the pedal, an elastic band was placed on the hemiparetic limb, involving the limb and the pedal. Two experienced physiotherapists supervised all training.

The dual-task exercise consisted of walking for 45 min and performing tasks such as: sitting/standing up while holding a ball, summing numbers, listening and remembering a story, saying the letters of the alphabet, repeating sentences, subtracting numbers in reverse, naming colors of flags, saying words that start with the last letter of a previous word, saying sentences in reverse, and naming fruits. These tasks were selected from previous studies (Kim G. Y. et al., 2014; Sasaki et al., 2015; Liu et al., 2017) and cover several cognitive functions such as attention, memory, language and executive functions (Kim G. Y. et al., 2014; Sasaki et al., 2015; Liu et al., 2017). Similar to the aerobic physical exercise group, participants in the cognitive dual-task gait group were encouraged to walk with weekly goals concerning the covered distance (Danks et al., 2014) as a way to break the vicious cycle of a sedentary lifestyle associated with stroke. Two physiotherapists and one occupational therapist guided the dual-task cognitive-gait sessions. In both groups, considering the installed conditions and the need to prevent adverse events, the intervention was provided to one participant at a time, depending on the order of arrival each day of the intervention. Both groups also benefited from 30 min of conventional physical therapy after each training session, which consisted of muscle-strengthening, balance, and gait activities like previous protocols (Liu et al., 2017).

In both groups, physical parameters such as height and body weight were evaluated immediately before the first training session. Blood pressure and oxygen saturation were measured before and after each training session to monitor participants’ health since low saturation values are often used as a criterion for stopping a stress test (Gaskin and Thomas, 1995). We did not record any incidents or worsening of the participants’ health status during the study.

To assess the presence of cognitive impairment that might compromise participation, we used the Mini-Mental State Examination (MMSE) cut-off point ≥19 (Yeh et al., 2021) before acute (T0) and after 12-week chronic exercise (T2). Attention and concentration performance was assessed at baseline (T0), post-acute (T1) and post-chronic intervention (T2) using the d2 Test of Attention (Brickenkamp, 2007; Maeneja et al., 2022).

**Figure 1:**
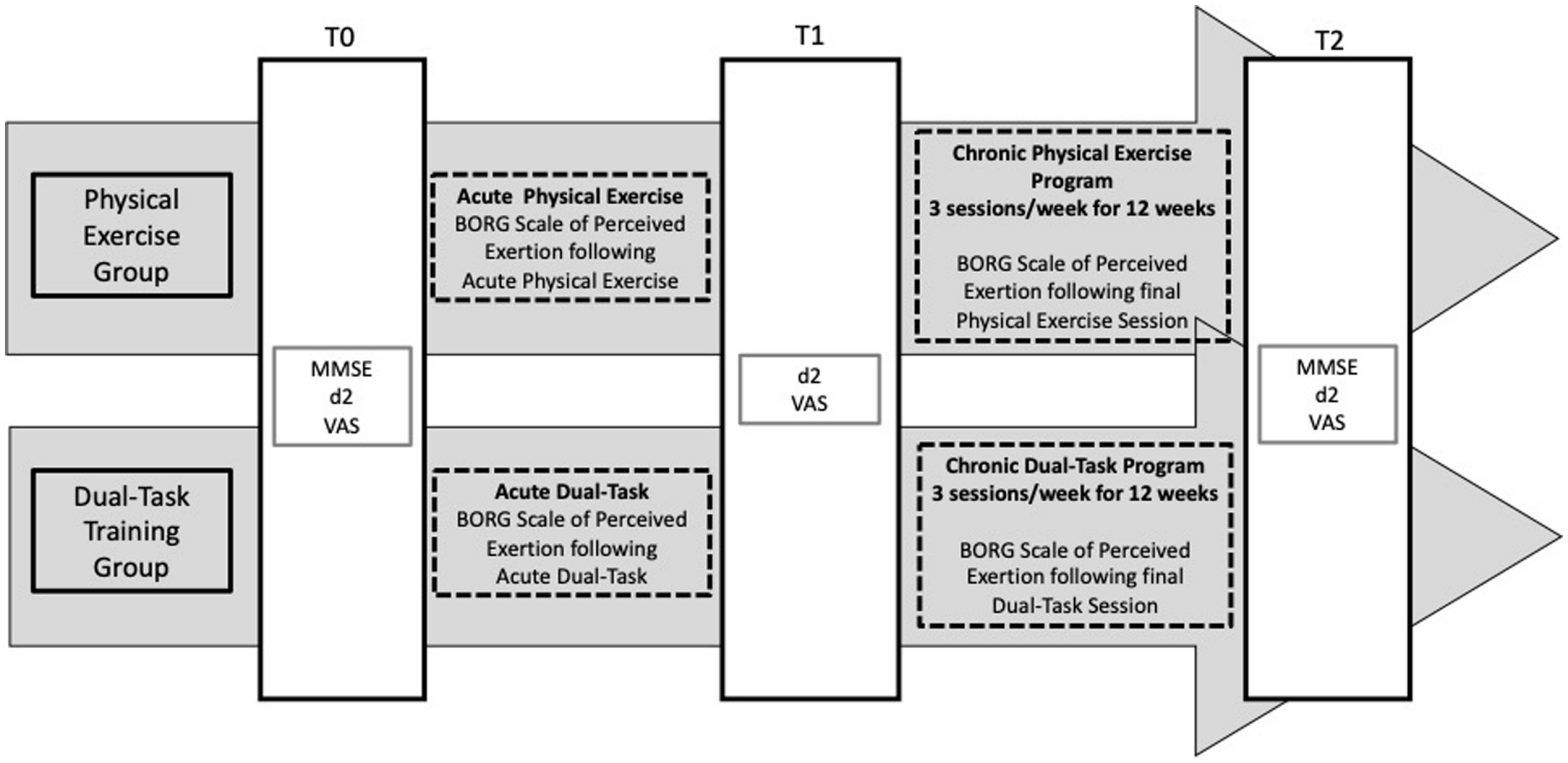
Research design timeline.

To assess participants’ perceived difficulty after 5 min of recovery from the task, a Visual Analog Scale (VAS) (Maeneja et al., 2022), was applied after the d2 Test of Attention. To assess participants’ perceived exertion, the Borg Perceived Effort Rating Scale (Borg, 1982; Aamot et al., 2014) was applied after the acute and last training session of each week throughout the 12-week chronic intervention protocol (Borg, 1982; Maeneja et al., 2022), as shown in Figure 1.

#### 2.3.2. Exercise safety and adherence

Before the start of the exercise, the participants learned to measure their level of effort according to the Borg rating of perceived exertion (RPE). Participants were able to rest whenever they exhibited signs of fatigue. During the 12 weeks of the intervention, participants were instructed to report any adverse events or effects.

Adherence was determined from sessions attendance and calculated as the percentage of total sessions attended. And the average number of sessions attended was 35 in both groups.

### 2.4. Study measures

#### 2.4.1. d2 test of attention

The d2 Attention Test is a timed, paper-and-pencil cancelation neuropsychological test that can measure individual attention and concentration performance, such as processing speed and quality of short-term performance (Siennicka et al., 2019; Woo et al., 2022). This test includes 14 lines, each consisting of 47 characters ‘p’ and ‘d’ with one to four strokes, arranged around the letters (Siennicka et al., 2019; Maeneja et al., 2022). The test presents three targets: the letter ‘d’ with two strokes above it, two strokes below it, or one stroke above and one below (da Silva-Sauer et al., 2022). The participant must go through each line to identify and cross out as many ‘d’ letters with two strokes as possible in a limited time of 20 s. The total test duration is 4 min and 40 s (Siennicka et al., 2019; Woo et al., 2022).

Evidence suggests that attention is one of the domains that is typically compromised with the onset of stroke (Cumming et al., 2013). Furthermore, it has been shown that attention and concentration improve with physical activity (Spitzer and Hollmann, 2013; Vanhelst et al., 2016; Reigal et al., 2020). Hence, in order to assess if chronic aerobic physical exercise might enhance attention and concentration, we chose the d2 test of attention, which was used before the start of the cognitive rehabilitation exercise program and after the end of the rehabilitation exercise program, as it measures these two processes, directly associated with the cognitive functions of our brain (Brickenkamp and Zilmer, 1998; Loetscher et al., 2019).

According to Brickenkamp (2007) and Steinborn et al. (2018), the d2 Test of Attention can be analyzed by assessing the total characters (TC), representing the number of processed characters, which informs about the speed with which the information is processed, specifically the speed of execution as well as productivity. Moreover, one can assess the total hits (TA) representing the number of characters marked correctly, which informs about the accuracy with which the task is performed. We were particularly interested in assessing the following domains: *General Efficiency score (TC-E)*, representing the total number of characters processed minus the total number of errors, which assesses attention control and inhibition, as well as the relationship between speed and meticulousness in performance, this result informs about the overall performance; *Concentration Index (CI)*, representing the total hits minus total omissions, which assesses the ability to concentrate by evaluating the combination between speed and accuracy; *Variability Index (IV)*, representing the difference between the maximum value (TC max) and the minimum value (TC min), which is an indicator of consistency in the execution of the task; and finally, *Error Percentage (E%)*, representing the percentage of errors made throughout the test, which is an indicator of meticulousness and quality of performance.

As referred above, here we were interested in attention and concentration. Thus, we assessed the following domains: General Efficiency score (TC-E), Concentration Index (CI), Variability Index (IV), and Error Percentage (E%), since with these results it is possible to obtain data on attentional control, ability to concentrate, stability and consistency in the execution of the task. Further, these domains afford the evaluation of the qualitative aspects of performance (Lindemann-Matthies et al., 2021; Maeneja et al., 2022).

#### 2.4.2. Mini-Mental State Examination

The Mini-Mental State Examination (MMSE) is a 30-point cognitive screening test including five categories, namely orientation, recording, attention and calculation, recall, and language (Molloy and Standish, 1997; Larner, 2020). It is an easy-to-apply test, completed in about 5 to 10 min, depending on the individual’s disability (Larner, 2020). Studies have shown that sociocultural variables, age, and education, can affect individual MMSE scores (Arevalo-Rodriguez et al., 2021). Given the level of education of the participants, wherein 29 of the 34 participants presented a basic schooling level, i.e., between 4 years and 10 years of schooling, we chose to use the Mental State Examination cutoff ≥19 (Paddick et al., 2015, 2017; Lestari et al., 2017; Yeh et al., 2021). Thus, in this study, the MMSE was used before starting the exercise program to screen eligible participants, as well as after the 12-week duration of the exercise program, with the aim of comparing cognitive performance in the pre and post exercise program, as MMSE is a widely used tool to measure cognitive impairment and detect changes over time (Canli and Ozyurda, 2020).

#### 2.4.3. Visual Analog Scale

The VAS was created in a clinical situation in order to measure pain perception (Ismail et al., 2015). It has since been used to assess perceived exertion after physical exercise (Tseng et al., 2010), mental effort (Pageaux and Lepers, 2018; Smith et al., 2019), the psychophysiological response in cognitive tasks (Fuentes-García et al., 2019), and perceived task difficulty (Ouwehand et al., 2021). Given that the participants experienced effort to perform the neuropsychological tests (MMSE and d2), we used the VAS to assess the perceived difficulty associated to this effort. It is possible that perceived difficulty may decrease for some participants, associated to the putative improvement in cognitive performance (Abd-Elfattah et al., 2015; Pageaux and Lepers, 2018; Smith et al., 2019). The Visual Analog Scale (VAS) measures a characteristic or attitude that on a continuum of values showing parametric properties (Dompeyre et al., 2007). It consists of a 100 mm straight line, with a (+) and (−) sign at each end, in which the participant draws a perpendicular line to identify their subjective state according to the research question (Delgado et al., 2018). The VAS is a simple pen and paper test, requiring only a sheet of paper with a printed line in the aforementioned dimension, a preceding question, and a plus and a minus symbol at either end of the line. The line can be horizontal or vertical (we chose vertical lines), as suggested by some authors for use in stroke (Ozyemisci-Taskiran et al., 2019).

Here we asked the participant to rate their perception of difficulty in the MMSE and d2 tasks, by bisecting the vertical line closest to the (+) or (−) symbols of the VAS (Johnson, 2001; Tseng et al., 2010; Delgado et al., 2018).

We used the VAS before starting the exercise program to assess the perception of difficulty in the cognitive tasks, in order to obtain data at baseline. Further, we used the VAS after the cognitive tasks subsequent to the acute exercise, and subsequent to the 12 week exercise program, with the aim of comparing the perception of difficulty in the pre- and post-exercise program (Shahar et al., 2019; Smith et al., 2019; Ouwehand et al., 2021).

#### 2.4.4. The Borg Scale

The Borg Scale or Borg Rating of Perceived Exertion (Borg, 1982) is a method for obtaining subjective estimates of exercise intensity and exertion and in both healthy and patient populations (Aamot et al., 2014), with scores ranging from 6 to 20. Perceived effort is defined as the ability to detect and respond to sensations that arise from the body during exercise (Pageaux, 2016; Cabral et al., 2020). The intensity of a training program is a critical variable in post-stroke treatment gains, and the Borg Scale is a valuable tool for measuring intensity after a stroke, as it is an easy-to-use scale that measures an individual’s self-rated physical exertion during exercise, which can even be accurately assessed by individuals with stroke, regardless of the severity of their motor impairments (Milot et al., 2019). To monitor the participants’ perceived exertion during the activity, the Borg Scale was used after the acute and last training session of each week throughout the 12-week chronic intervention protocol as in a previous study (Maeneja et al., 2022), since the physical fatigue that occurs during physical exercise seems to influence the specific performance of the task, as well as impair performance in tasks that require target detection (Moore et al., 2012). The development of perceived exertion involves numerous neural processes occurring in various regions of the brain, wherein efferents from the central unit are sent from the motor to the sensory regions of the brain, and therefore perceived exertion is considered extremely important in regulating intensity during physical activity (Abbiss et al., 2015). Specifically, during the rehabilitation of people with stroke, exercise intensity is often guided by subjective reports using surrogate measures such as the Borg Scale of Rating of Perceived Exertion (Spring, 2016).Thus, the Borg Scale was used to assess the intensity of exercise from the perspective that it was safe, allowing participants to acquire cardiovascular fitness little by little, thus avoiding adverse events, as it is known that post-stroke individuals have less than half of the cardiorespiratory fitness (Crozier et al., 2018).Therefore, the assessment of subjective perception of effort is essential during physical work, as the symptom of exertion is unique to an individual and can be used as a subjective estimate of physical intensity (Williams, 2017). We would assume that, as the participants rehabilitation progresses, perception of effort should decrease allowing for an increase in physical intensity.

### 2.5. Statistical analysis

Based on previous studies (Billoir et al., 2015), we calculated the sample size, taking into account a previous study of 20 participants conducted by Kim G. Y. et al. (2014), where the data analyses were performed using SPSS, wherein the normal distribution of the data was confirmed using the Shapiro–Wilk test, and two-way repeated measures ANOVA was also performed to compare changes in cognition between two groups. In this study by Kim G. Y. et al. (2014), the measure of the effect size was large, and therefore we considered an effect size F of 0.46 which corresponds to a large effect. On the other hand, resorting to simulation approaches (Billoir et al., 2015), we calculated on *a priori* number of participants needed to reach sufficient power using a free software G * Power (Faul et al., 2007, 2009). For an ANOVA statistical test: repeated measures, intermediate interaction, with an effect size of 0.25 (medium effect), a significance level of 0.05, and an observed power of 0.80, we determined a sample of 28 participants. A more conservative sample of 57 participants was engaged to control for experimental mortality and attrition. The analysis followed the intention-to-treat principle (Best et al., 2018), as all randomized participants were included to estimate treatment effects. All participants were engaged in the treatment up to the end of the experiment.

In order to verify the existence of significant differences between the two groups (aerobic physical exercise/dual task exercise) in the sociodemographic variables, inferential statistics were used. The non-parametric Chi-square test was used to compare the two groups regarding gender, since this variable has a nominal scale, and the Fisher test was used to compare the two groups by type of stroke and education, since there was more than 20% of cells with an expected count less than 5. A parametric Independent Sample *t*-test was also used to compare the two groups in quantitative variables (age, weight, height and MMSE results). These parametric statistical tests were chosen since the distribution was normal (Shapiro test) or presented negligible deviations from normality (SK from −3 to 3 and KU from −7 to 7) according to Marôco (2018). To investigate the primary outcomes, we used Mixed-Model ANOVAs. A mixed model ANOVA, is appropriate when examining for differences in a continuous level variable by group and time. The primary purpose of a mixed ANOVA is to understand if there is an interaction between these two factors on the dependent variable. The independent variable of this study is the type of exercise (aerobic physical exercise /dual-task exercise) and the dependent variable are: Attention, General Efficiency, Concentration Index, Variability Index, Error Percentage. To investigate the secondary outcomes, we also used mixed-model ANOVAs. As we mentioned earlier, the independent variable is the type of exercise (aerobic physical exercise/dual task exercise), and in this case, the secondary dependent variables are: perceived task difficulty measured by VAS and perceived effort measured by the Borg Scale. These are particularly useful as they provide supporting evidence for the primary outcome (Vetter and Mascha, 2017).

These parametric statistical tests were chosen since the distribution was normal or had a less severe deviations from normality (SK from −3 to 3 and KU from −7 to 7), and presenting sphericity (Mauchly test) in the Within ANOVAs and homogeneity of variances (Levéne test) in the Between ANOVAs. We did not include any covariates in this analysis since the groups are similar in sociodemographic variables. A measure of effect size of the was also used (partial eta square effect). The following rules of thumb are used to interpret values for Partial eta squared: 0.01: Small effect size, 0.06: Medium effect size 0.14 or higher: Large effect size. We complemented the mixed ANOVA with one-way ANOVAs and Eta partial square to detect at which moments there are significant differences between the 2 groups and with ANOVAs for repeated measures to see the evolution within each group over the 3 moments. These analyses thus allow a better understanding of the interaction effect.

## 3. Results

### 3.1. Participant characteristics

**Figure 2:**
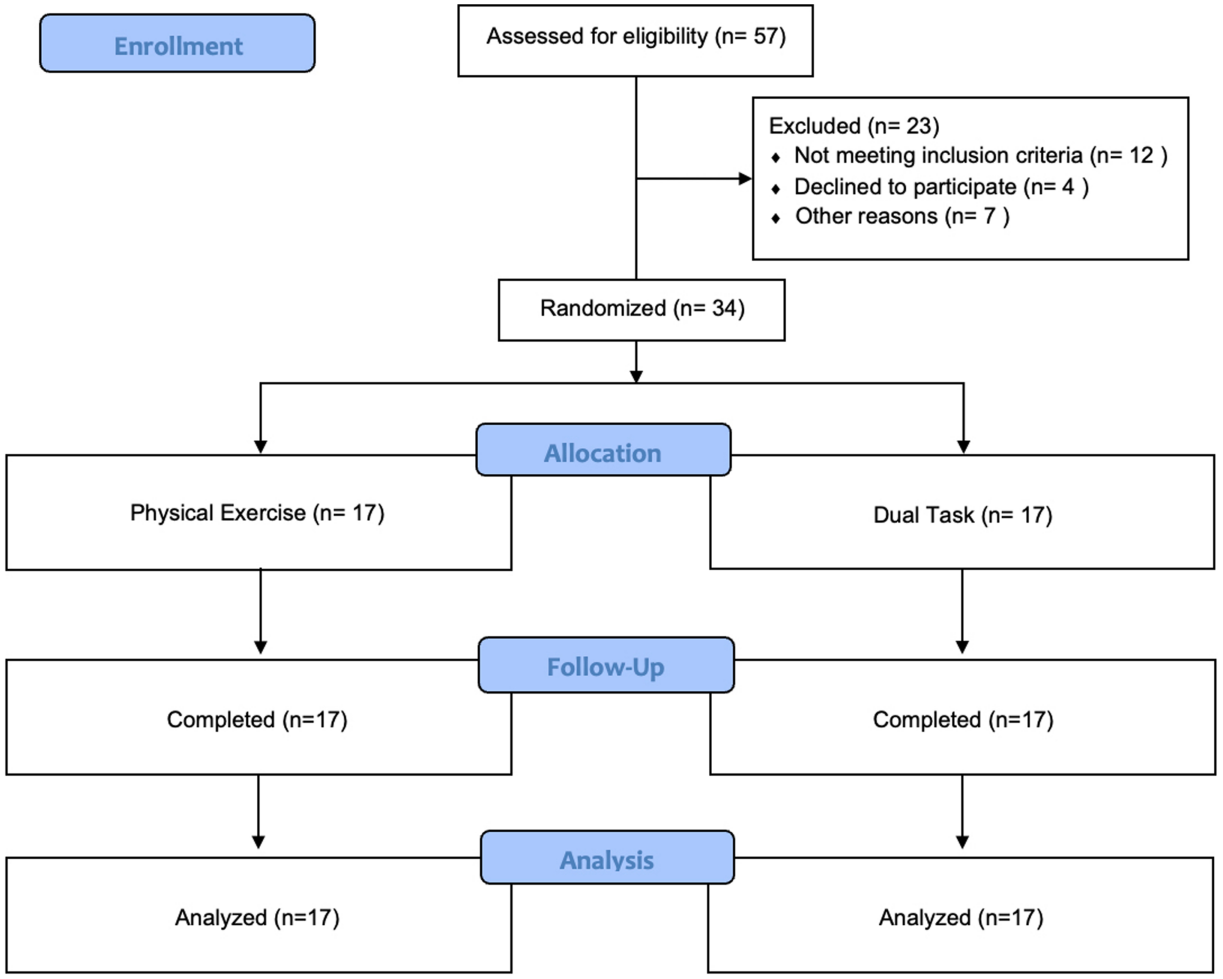
Flow diagram.

Recruitment took place between July 2021 and May 2022. A total of 34 patients were included in the final analysis in this randomized clinical trial with blinded participants. The study ended when data collection for all recruited participants was complete (Aug 31,2022). Seventeen participants were assigned to each group as shown in Figure 2. As shown in Table 1, the PE and DT training groups were similar concerning baseline characteristics including demographics (age, gender, and education level), weight, and cognitive outcomes on MMSE. In both PE and DT groups, we find a slightly superior percentage of men compared to women (52.9% in PE and 58.8% in DT). In both groups, most participants had been enrolled in basic schooling (up to 10 years of school) (94.1% in PE and 76.5% in DT). The only variable presenting a significant difference between groups is height (*p* = 0.039), with the PE group with a mean height of 1.63 m and the DT group slightly taller with a mean height of 1.65 m. Although not expressive, the differences in the ratio between men and women amongst the two groups might subtend the difference in height in these same groups.

**Table 1:** Sociodemographic characterization of the PE and DT groups concerning gender, age, weight, height, MMSE at baseline, and schooling.

	PE (*n* = 17)	DT (*n* = 17)	
Gender			
Women	47,0.1% (8)	41.2% (7)	*X*^2^ = 0.119, *p* = 0.730
Men	52.9% (9)	58.8% (10)	
Schooling			
Basic	94.1% (16)	76.5% (13)	Fisher =3.153, *p* = 0.227
Intermediate	0.0% (0)	17.6% (3)	
Superior	5.9% (1)	5.9% (1)	
Age	Mean = 55.12 Std =6.660	Mean = 57.00 Std =10.23	*t* = −0.354 *p* = 0.726
Weight	Mean = 71.45 Std =11.44	Mean = 71.85 Std =9.06	*t* = 0.278 *p* = 0.783
Height	Mean = 1.63 Std =0.05	Mean = 1.65 Std =0.11	*t* = −2.203 *p* = 0.039
MMSE 0	Mean = 25.18 Std =2.69	Mean = 25.29 Std =2.93	*t* = 1.186 *p* = 0.244

*X*^2^, Chi square; *t*, independent samples *t*-test.

### 3.2. Primary outcomes

#### 3.2.1. Cognitive performance pre- to post-intervention

##### 3.2.1.1. d2 test of attention

To assess possible differences on the principal domains of the d2 test of attention, between the PE and DT groups from pre- to post-intervention, we computed several ANOVAs within and between factors.

**Figure 3:**
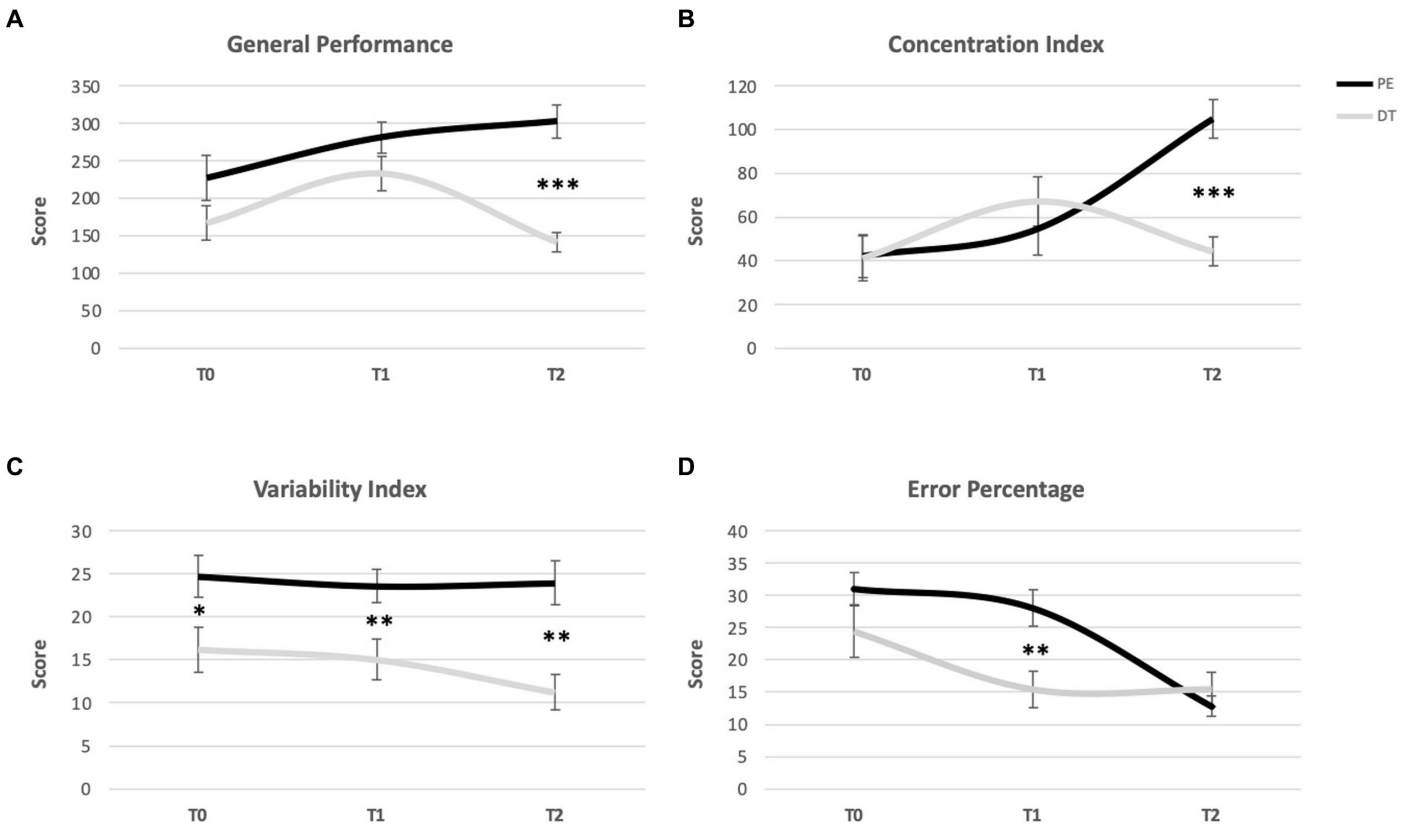
Distribution of the mean score over time (from T0 to T1 and from T1 to T2,) in different d2 domains: **(A)** General Efficiency; **(B)** Concentration Index; **(C)** Variability Index; and **(D)** Error Percentage. * indicates significant differences for *p* < 0.05; **Indicate significant differences for *p* < 0.01; and ***Indicate significant differences for *p* < 0.001. Error bars represent standard errors. Only Between group differences are portrayed. For differences Within groups, please see Table 2.

The results regarding d2 are presented in Table 2. For the *General Efficiency* score Mixed-Model ANOVA revealed a significant interaction effect with a high large effect size [*F*(2) = 8.499, *p* = 0,001, ηp^2^ = 0.210] and observed power = 0.959. There were no significant differences between groups at T0 and T1, with a significant difference for T2 and a large effect (ηp^2^ = 0,552) with the PE group showing a higher performance (*M* = 302.41; SD = 90.68) compared to the DT group (*M* = 141.29; SD = 54.37). Considering performance over time, in the PE group there was a significant improvement from T0 to T2 (*p* = 0.006), with a significant difference from T0 to T1 and stabilization from T1 to T2; in the DT Group, there were no significant differences between T0 to T2 (*p* = 0.317), with an increase from T0 to T1 and decrease from T1 to T2 (Figure 3A).

**Table 2:** ANOVA tests of within and between effects for d2 domains.

D2 Domains		T0		T1		T2		Tests of Within Effects
		Mean	Std. Deviation	Mean	Std. Deviation	Mean	Std. Deviation	
General performance	PE (*n* = 17)	226.71	124.05	281.00	84.15	302.41	90.68	*F* = 7.772 *p* = 0.002** *Post Hoc*: T0 vs. T1 *p* = 0.018* T0 vs. T2 *p* = 0.006** T1vs T2 *p* = 0.143
	DT (*n* = 17)	167.06	96.28	233.53	93.91	141.29	54.37	*F* = 8.683 *p* = 0.001*** *Post Hoc*: T0 vs. 1 *p* = 0.006** T0 vs. 2 *p* = 0.317 T1vs 2 *p* = 0.001***
	Tests of between effects	*F* = 2.453 *p* = 0.127		*F* = 2.410 *p* = 0.130		*F* = 39.473 *p* = 0.000***		
Concentration index	PE (*n* = 17)	41.94	39.84	54.41	48.79	104.76	36.72	*F* = 23.728 *p* = 0.000*** *Post Hoc*: T0 vs. T1 *p* = 0.096 T0 vs. T2 *p* = 0.000*** T1vs T2 *p* = 0.000***
	DT (*n* = 17)	41.24	43.35	67.12	46.17	44.29	26.63	*F* = 5,037 *p* = 0,013* *Post Hoc*: T0 vs. T1 *p* = 0.001*** T0 vs. T2 *p* = 0.746 T1vs T2 *p* = 0.04*
	Tests of between effects	*F* = 0.002 *p* = 0.961		*F* = 0.608 *p* = 0.441		*F* = 30.216 *p* = 0.000***		
Variability index	PE (*n* = 17)	24.71	9.90	23.59	8.03	23.94	10.63	*F* = 0.064 *p* = 0.938 *Post Hoc*: T0 vs. T1 *p* = 0.627 T0 vs. T2 *p* = 0.839 T1 vs. T2 *p* = 0.919
	DT (*n* = 17)	16.18	10.98	15.00	9.78	11.24	8.57	*F* = 1.296 *p* = 0.288 *Post Hoc*: T0 vs. T1 *p* = 0.641 T0 vs. T2 *p* = 0.196 T1vs T2 *p* = 0.279
	Tests of between effects	*F* = 5.657 *p* = 0.024*		*F* = 7.830 *p* = 0.009**		*F* = 30.216 *p* = 0.001**		
Error percentage	PE (*n* = 17)	31.04	10.51	28.06	11.46	12.78	6.50	*F* = 23.769 *p* = 0.000*** Post Hoc: T0 vs. T1 *p* = 0.248 T0 vs. T2 *p* = 0.000*** T1vs T2 *p* = 0.000***
	DT (*n* = 17)	24.44	16.62	15.36	11.54	15.37	11.09	*F* = 3.295 *p* = 0.041* Post Hoc: T0 vs. T1 *p* = 0.005** T0 vs. T2 *p* = 0.007* T1vs T2 *p* = 0.999
	Tests of between effects	*F* = 1,915 *p* = 0,176		*F* = 10.368 *p* = 0,003**		*F* = 0,693 *p* = 0,411		

*p < 0.05; **p < 0.01; ***p < 0.001.

As for the *Concentration Index* a Mixed-Model ANOVA revealed a significant interaction effect with a large effect size [*F*(2) = 17,580, *p* = 0,000, ηp^2^ = 0,355] and an observed power = 1.00.

There were no significant differences between groups at T0 and T1, with a significant difference at T2 and a large effect (ηp^2^ = 0,486), with the PE group presenting a higher performance (*M* = 104.76; SD = 36.72) compared to the DT group (*M* = 44.29; SD = 26.63). Considering performance over time, in PE group there was a consistent improvement from T0 to T2 (*p* = 0.000), while such increase was not observed in the DT Group (*p* = 0.746) (Figure 3B).

Concerning the *Variability Index* Mixed-Model ANOVA revealed no significant interaction effect [*F*(2) = 0,561, *p* = 0,573, ηp^2^ = 0,017]. There were significant differences between the groups in the three assessment moments, with the PE group having higher averages [despite performance within each group not showing a significant evolution (Figure 3C)].

Regarding *Error Percentage* a Mixed-Model ANOVA revealed a significant interaction effect [*F*(2) = 4.797, *p* = 0.011, ηp^2^ = 0.130] and a medium effect size, there were no significant differences between the two groups at T0 (*p* = 0.176) and T2 (*p* = 0.411), with a significant difference *p* < 0.01 at T1 and a large effect size (*p* = 0.003, ηp^2^ = 0.150), with the PE group presenting a higher result in Error Percentage (mean = 28.06), compared to the DT group (mean = 15.36). Regarding evolution over time within each group, in the PA Group there were no significant differences from T0 to T1 (*p* = 0.248), with an extremely significant increase in the result from T1 to T2 (*p* = 0.000), and an extremely significant difference between T0 and T2 (*p* = 0.000) as well. In the DT Group, the result decreases significantly from T0 to T1 (*p* = 0.005), and remains stable from T1 to T2 (*p* = 0.999), with a significant difference between T0 and T2 (*p* = 0.007) (Figure 3D).

#### 3.2.2. MMSE

A Mixed-Model ANOVA revealed a significant interaction effect [*F*(1) = 43.267, *p* = 0.000, ηp^2^ = 0.575] and a large effect size. As desirable, MMSE test results of did not differ in T0 (M_PE_ = 25.18 vs. M_DT_ = 25.29; *p* = 0.904) showing no differences between groups pre-intervention, and a small effect size. However, in T2, PE and DT present an extremely significant difference (*p* = 0.000), and a large effect size, with the PE group increasing its mean score (*M* = 27.12) and the DT group decreasing its mean score (*M* = 23.82) as shown in Table 3; Figure 3.

**Table 3:** MMSE: intra- and inter-group comparisons.

	MMSE_0		MMSE_2		
	Mean	Std. deviation	Mean	Std. deviation	Tests of within effects
PE (*n* = 17)	25.18	2.70	27.12	1.27	*F* = 19.360 *p* = 0.000**
DT (*n* = 17)	25.29	2.93	23.82	2.51	*F* = 29.070 *p* = 0.000***
Tests of between effects	*F* = 0.015 *p* = 0.904		*F* = 23.381 *p* = 0.000***		

*p < 0.05; **p < 0.01; ***p < 0.001.

### 3.3. Secondary outcomes

#### 3.3.1. Perceived difficulty and perceived exertion pre- to post-intervention

##### 3.3.1.1. Visual Analog Scale

**Figure 4:**
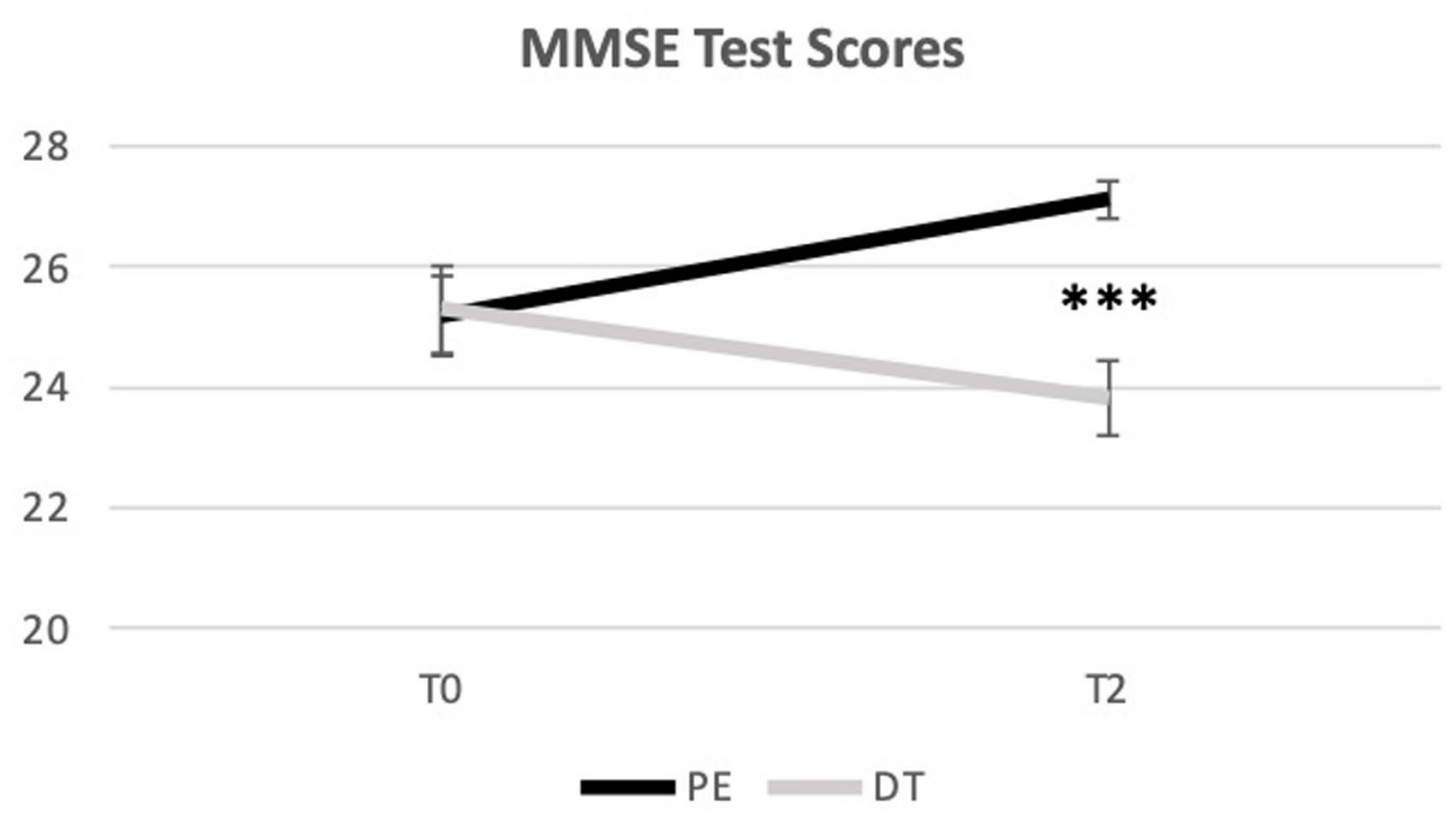
Distribution of the MMSE mean score over time (from T0 to T2). ***Indicate significant differences for *p* < 0.001. Error bars represent standard errors. Only Between group differences are portrayed. For differences Within groups, please see Table 3.

**Figure 5:**
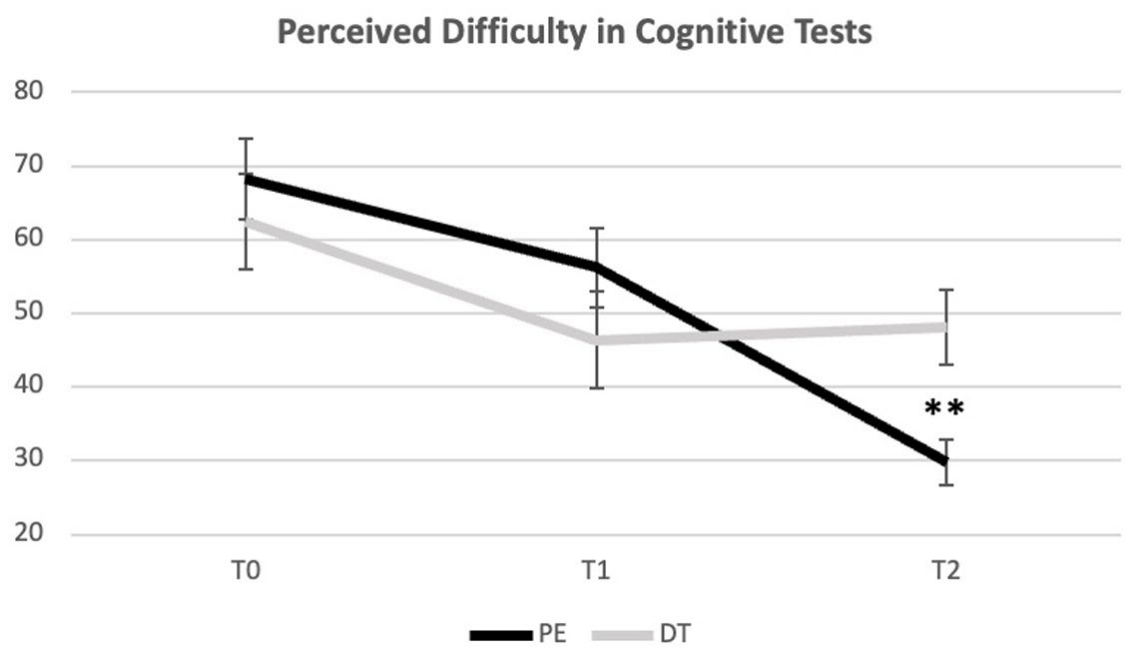
Evolution of perceived difficulty in cognitive tests (d2 and MMSE in T0 and T2; d2 in T1) across time points. **Indicate significant differences for *p* < 0.01. Error bars represent standard errors. Only Between group differences are portrayed. For differences Within groups, please see Table 4.

Regarding the VAS applied after the d2 Test of Attention, a Mixed-Model ANOVA revealed a significant interaction effect [*F*(2) = 6.190, *p* = 0,003, ηp^2^ = 0.161] and a large effect size. There were no significant differences between PE and DT in T0 and T1. However, we observed a significant difference in T2 (*p* = 0.004), having the PE group decreased perception of difficulty (*p* = 0.004) in the cognitive tasks (d2 and MMSE) (*M* = 2.98; SD = 1.26), compared to the DT group (*M* = 4.81; SD = 2.08) (Figures 4, 5; Table 4).

**Table 4:** ANOVA tests of within and between effects for the VAS score.

	VAS_0		VAS_1		VAS_2		
	Mean	Std. deviation	Mean	Std. deviation	Mean	Std. deviation	Tests of within effects
PE (*n* = 17)	6.83	2.25	5.62	2.20	2.98	1.26	*F* = 22.842 *p* = 0.000*** Post Hoc: T0 vs. T1 *p* = 0.055 T0 vs. T2 *p* = 0.000*** T1vs T2 *p* = 0.000***
DT (*n* = 17)	6.24	2.66	4.64	2.72	4.81	2.08	*F* = 3.741 *p* = 0.035* Post Hoc: T0 vs. T1 *p* = 0.060 T0 vs. T2 *p* = 0.043* T1vs T2 *p* = 0.648
Tests of between effects	*F* = 0.495 *p* = 0.487		*F* = 1.343 *p* = 0.255		*F* = 9.629 *p* = 0.004**		

*p < 0.05, **p < 0.01, ***p < 0.001.

##### 3.3.1.2. Borg rating scale of perceived exertion

**Figure 6:**
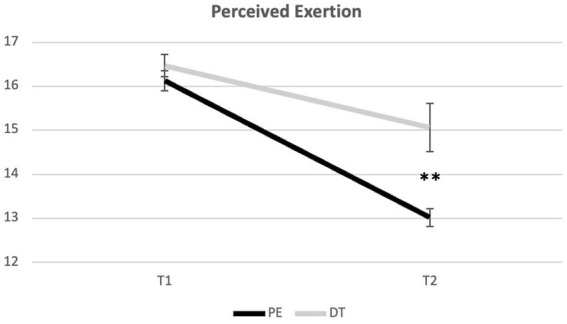
Evolution of perceived exertion across time points. **Indicate significant differences for *p* < 0.01. Error bars represent standard errors. Only Between group differences are portrayed. For differences within groups, please see Table 5.

Regarding the Borg rating scale, Mixed-Model ANOVA revealed a significant interaction effect [*F*(1) = 6.963, *p* = 0.013, ηp^2^ = 0.179] and a large effect size. Concerning perceived exertion, the groups did not show significant differences after acute training but did so at the end of the intervention program (*p* = 0.001), with the DT group presenting a higher average (*M* = 15.07; SD = 2.26) compared to the PE group (*M* = 13.02; SD = 0.84) (Table 5; Figure 6).

**Table 5:** BORG: intra- and inter-group comparisons.

	BORG_1 acute		BORG_2 chronic		
	Mean	Std. deviation	Mean	Std. deviation	Tests of within effects
PE (*n* = 17)	16.12	0.93	13.02	0.84	*F* = 151.308 *p* = 0.000**
DT (*n* = 17)	16.47	1.07	15.07	2.26	*F* = 5.605 *p* = 0.031*
Tests of between effects	*F* = 1.059 *p* = 0.311		*F* = 12.33 *p* = 0.001**		

*p < 0.05, **p < 0.01, ***p < 0.001.

## 4. Discussion

A recent review that examined randomized clinical trials reports inconclusive evidence of the beneficial effect of exercise interventions exercise on cognitive function (Ciria et al., 2023). Here we aimed to assess whether aerobic physical activity influences post-stroke cognitive recovery, namely on attention and concentration functioning. That is, if aerobic physical exercise presents a distinct impact on cognition compared to the traditional dual-task intervention in patients with stroke. We constituted two groups, with one performing aerobic physical exercise and the other performing dual-task cognitive-gait training, the two groups were equivalent in sociodemographic characteristics such as age, gender, and education. For participant cognitive screening, we used the MMSE which showed that the participants were matched at baseline.

Furthermore, the literature explains that dual tasks include a controlled combination of two tasks or activities performed simultaneously (Netz, 2019), and that its practice reduces the interference between dual tasks over time (Strobach and Schubert, 2017). Here, the PE group consistently presented higher cognitive gains compared to the DT group. Our results showed that cognitive performance of patients with stroke was significantly different after the two types of interventions: aerobic physical exercise and dual-task gait exercise. The results corroborate that in patients with stroke, physical exercise training leads to greater cognitive gains when compared to dual-task gait exercise, with evident improvements after 12 weeks of chronic exercise.

We showed that there are differences in the General Efficiency score over time in the PE group, while in the DT group there was no significant improvement. There was a significant reduction in attention errors after the intervention in the PE group. The decrease in scores observed from T1 to T2, suggests that the DT was not only less effective but even seemed to have been detrimental. It is possible that this might result from therapeutic saturation (Ahmed et al., 2021) that explains that repetitive exercises cease to be challenging, especially from the intensity point of view (Page et al., 2004; Ciria et al., 2023). Another possibility might consist in the so-called “principle of posture first’ where, in challenging tasks (dual-task), there is a tendency to prioritize the motor task to the detriment of the cognitive task (Timmermans et al., 2018). This causes cognitive performance to decrease significantly (Smulders et al., 2012), in association with low motivation, since the tasks introduced by the dual-task were not new, may not have corresponded to the expectations of the participants, and consequently, might not have been motivating. As it is well known, a person with low expectations will have a more internal focus and lower performance (Machan and Krupps, 2021). Therefore, premorbid low levels of cardiorespiratory fitness that likely contribute to post-stroke deconditioning (Billinger et al., 2015), require aerobic exercise at high enough levels to improve cardiorespiratory fitness to promote plasticity (Ploughman and Kelly, 2016). As for the Concentration Index, in the PE group there was a consistent improvement from T0 to T2, while such an increase was not observed in the DT group. This sustains that PE has a superior benefit on concentration. As for the Variability Index, there were significant improvements between the groups across the three moments of evaluation, which points to the possibility that both PE and DT have similar contributions to the consistency of performance. Finally, in relation to the Error Percentage, we noticed a significant decrease in errors post-acute intervention for DT, with stabilization throughout chronic intervention. In the PE group the decrease in error percentage occurred only post-chronic intervention with PE and DT groups ending up with similar error percentages at T2. Thus, there was an increase in meticulousness and quality of performance occurring at different time-points but evolving to the same endpoint. Together, these data show that PE seems to show greater benefits across cognitive domains compared to DT and that the gains in attention and concentration occur in chronic exercise and not exactly in acute exercise, and this is consistent with the fact that relatively long rehabilitation training programs tend to be more effective (Bernhardt et al., 2017; Bo et al., 2019).

These results translate into an improvement in selective and sustained attention, which is in line with other investigations in patients with traumatic brain injury (Blanchet et al., 2016; Romanov et al., 2021).We observed similar results in the MMSE, with a significant improvement in the score between the baseline and post-intervention, and greater significance for the PE group. These results are consistent with previous studies (Fernandez-Gonzalo et al., 2016; Yeh et al., 2021; Liu-Ambrose et al., 2022) and emphasize the influence of physical exercise on improving post-stroke cognitive performance (Chan and Tsang, 2017; Barha et al., 2019; Yeh et al., 2019; Zheng et al., 2020).

The possible motive for the higher cognitive improvement in the PE compared to the DT task might be due to the low motor intensity applied to walking in DT, given the walking difficulties that usually accompany stroke survivors prevented the implementation of a walking pace that would help promote relevant physiological changes (MacKay-Lyons et al., 2020; Yang and Wang, 2021) (in PE the stride was aided by the use of the treadmill), also associated with the fact that in the face of challenging walks (dual-task), a very common adaptation is to walk slower (Timmermans et al., 2018). However, this reflection around the possible reason for the greater cognitive improvement in the PE group compared to the DT task seems confusing, especially when there is a higher RPE (*M* = 15.07) in the DT group compared with the PE group (*M* = 13.02). These data have an explanation, as the literature reports that RPE is greater when performance involves a cognitive component (Condello et al., 2019). Further, in this protocol we compare two different exercise modalities, it is natural that the RPE is different, as there is evidence that indicates that perceived exertion is associated with the type of exercise (Andrade et al., 2020; Herold et al., 2020; Lea et al., 2022).

Furthermore, since dual-task interference occurs between 40 and 60% of motor intensities (Kimura et al., 2022), when gait performance is low there is also low performance on neuropsychological measures (Blumen et al., 2014). Therefore, intensity is a factor that drives affective responses to exercise (Jones and Zenko, 2021), as well as associated neural changes and cognitive outcomes (Dhir et al., 2021). A systematic review by Lauenroth et al. (2016), also highlights the importance of intensity, by explaining that an important factor to consider when choosing the type of training, is the intensity of the exercise, as for an intervention program to be effective, it needs an increasing level of difficulty (Lauenroth et al., 2016) to optimize the use of an individual’s latent potential (Law et al., 2014).

Regarding the significant improvements in cognition after the sequenced aerobic and resistance physical activity program (PE group) on the treadmill, stationary bike, and desk pedal exerciser, we believe that this program was motivating from the perspective of novelty, since those training devices were novel to all participants (Parfitt et al., 2012; Wulf and Lewthwaite, 2016; Marcos-Pardo et al., 2018; Lakicevic et al., 2020; Schättin et al., 2021). In addition, the PE condition helped promote generalized biological changes of a rhythmic nature in cardiorespiratory rate, muscle activation time, and brain activity (Combourieu Donnezan et al., 2018; Tomporowski and Qazi, 2020), as the rhythm imposed by the machine regularizes the gait of the person with stroke, with increasingly longer and less variable steps, which generates positive effects on the central nervous system (Borrione, 2014; Penati et al., 2019). Therefore, unlike cognitive training which is designed to have specific effects and improve functioning in the neurocognitive domain, the impact of exercise, on brain health and cognition are broad and non-specific (Dhir et al., 2021). The cardiovascular fitness imposed by this type of program (aerobic and resistance physical exercise) is shown to have sparing effects on the prefrontal, superior parietal, and temporal cortices; as it affects cerebral blood flow, increases capillary density, cerebral angiogenesis, causes neurotrophic stimulation, increases plasticity of neurotransmitter systems, and increases white matter volume in the prefrontal cortex, hippocampus, and cerebellum and motor cortex with global cognitive implications (Netz, 2019; Williams, 2021).

Furthermore, it is possible that the cognitive gains observed in the PE group led to the decrease in perceived difficulty in the cognitive task. This might be due to the actual increase in attention and concentration, affording a more focused performance leading to the perception of decreased difficulty (Blanchet et al., 2016; Reigal et al., 2020). Conversely, previous studies (Marino et al., 2009; Ouwehand et al., 2021) have shown that subjective perception of cognition is more related to mood than to performance. Given that mood benefits from even a single bout of exercise as early studies suggest (Yeung, 1996), it is possible that the decrease in perceived difficulty in the cognitive tasks might be an indirect consequence of the PE mechanisms. Indeed, exercise affects mood through certain psychosocial mediators such as self-efficacy, self-esteem, decreased stress and anxiety levels (Chan et al., 2018), as well as through neurophysiological adaptations in the nervous system, such as elevation of body temperature that activates the sympathetic nervous system and the hypothalamic–pituitary–adrenal hormonal axis, and the hormonal changes that result in reduced pain perception and improved mood, increased endorphin levels and neurotransmitter production (Mikkelsen et al., 2017). The direct benefits of PE such as cardiovascular conditioning (Chapman et al., 2013; Netz, 2019) and increase in arousal (Gallotta et al., 2015; Ouwehand et al., 2021) might affect perceived exertion. The PE group decreased perception of exertion compared to the DT group suggesting that the intervention also contributed to increased fitness of this group.

Our results, along with evidence from the literature (Desjardins-Crepeau et al., 2016; Bo et al., 2019; Baek et al., 2021a; Kimura et al., 2022), suggest that both a combination of exercise (e.g., aerobic and strength exercise, or aerobic, strength and endurance) as well as dual task-based cognitive intervention are beneficial for post-stroke cognitive rehabilitation. This occurs since aerobic physical exercise affects global cognition through improved cardiovascular conditioning, while motor training is task specific in increasing brain neuroplasticity, affecting cognition (Netz, 2019). However, due attention must be paid to exercise intensity for relevant physiological changes to occur (Howard-Jones, 2014; MacKay-Lyons et al., 2020; Yang and Wang, 2021). Despite extensive literature showing the benefits of both dual task and combined exercise, there are studies that question the improvements attributed to dual task components, arguing that this type of training has limited transferability (Law et al., 2014; Embon-Magal et al., 2022). Others further claim that it is unclear whether Dual Task skills can be transferred into new Dual Task contexts and maintained over time (Joubert and Chainay, 2018; Lemke et al., 2018). Physical exercise, on the other hand presents evidence that its effects transfer to untrained contexts (Dhir et al., 2021; Kardys et al., 2022) given that the neural mechanisms responsible for the impact of physical training on cognition stem from neurogenesis (i.e., production of new neurons), angiogenesis (i.e., growth of new blood vessels from existing ones), synaptogenesis (i.e., formation of synapses between neurons), and the action of neurotrophins (proteins that support the survival, development, and functions of neurons) (Liu-Ambrose et al., 2008; van Praag, 2009; Joubert and Chainay, 2018; Dhir et al., 2021).

Importantly, our initial expectation that post-stroke physical activity would lead to greater cognitive gains compared to post-stroke dual-task cognitive walking was supported by our results after 12 weeks intervention. Clinicians and technical staff should be aware of the heightened benefits of aerobic exercise, not only for attention and concentration, but also for perception of difficulty and perceived exertion concerning the intervention *per se* in post-stroke cognitive rehabilitation. The benefits of dual-task cognitive walking are supplanted by simpler, more engaging aerobic exercise protocols.

## 5. Limitations

This work was not without limitations. First, we recruited patients with subacute to chronic ischemic stroke. This means that we obtained a heterogeneous sample of participants, including people in the process of cognitive and neurofunctional rehabilitation and people that have already stabilized concerning cognitive and functional processes. In the future, it is important to address this issue. Second, the use of the MMSE for cognitive screening has been considered outdated by some researchers, e.g., (Carnero-Pardo, 2014). However, we were limited by the instruments available at the participating institutions. In the future, more effective and inclusive short cognitive screening tests should be considered. Third, the fact that the intervention took place in parallel in two different places, combined with the trust placed in the evaluators (experienced health professionals) contributed to the non-evaluation of reliability in the terms defined by Hoffmann et al. (2014), as the degree to which an intervention occurred as intended. Finally, the comparison of two exercise modalities (PE vs. DT), without control, might also constitute a limitation. Specifically, to determine the strength of the aerobic physical exercise training, a control group with conventional physiotherapy would have been necessary. In the future, to better understand the efficiency of aerobic physical exercise, these aspects should be considered accordingly.

## 6. Conclusion

Cardiovascular skills through aerobic physical exercise reduce cognitive deficits, as cardiovascular capacity contributes to the improvement of cognitive performance. Aerobic physical exercise promotes angiogenesis in areas of the brain that were previously ischemic as a result of improved cardiovascular competence (Bliss et al., 2021; Zhou et al., 2021).

With this study we were able to show that the practice of aerobic physical exercise is associated with improvements in cognitive performance in patients with stroke. In addition, we showed that the perception of cognitive difficulty and physical exertion decrease with chronic aerobic physical exercise. Therefore, this study confirmed that the benefits for cognitive rehabilitation obtained through the practice of aerobic physical exercise seem to be relevant and can be recommended as a complementary rehabilitation to improve cognitive functioning in patients with stroke.

## Data availability statement

The datasets presented in this study can be found in online repositories. The names of the repository/repositories and accession number(s) can be found at: https://osf.io/c2hd4/?view_only=4c2326f8714241beb8b7fc3b903e24ac.

## Ethics statement

The studies involving humans were approved by Comissão de Ética para a Saúde (CES), UCP. The studies were conducted in accordance with the local legislation and institutional requirements. The participants provided their written informed consent to participate in this study.

## Author contributions

RM: Conceptualization, Investigation, Writing – original draft. CS: Data curation, Formal analysis, Writing – review & editing. IF: Methodology, Writing – review & editing. AA: Conceptualization, Funding acquisition, Methodology, Project administration, Resources, Supervision, Validation, Visualization, Writing – review & editing.
